# Inhibition of mTOR by Rapamycin Abolishes Cognitive Deficits and Reduces Amyloid-β Levels in a Mouse Model of Alzheimer's Disease

**DOI:** 10.1371/journal.pone.0009979

**Published:** 2010-04-01

**Authors:** Patricia Spilman, Natalia Podlutskaya, Matthew J. Hart, Jayanta Debnath, Olivia Gorostiza, Dale Bredesen, Arlan Richardson, Randy Strong, Veronica Galvan

**Affiliations:** 1 Department of Physiology, University of Texas Health Science Center at San Antonio, San Antonio, Texas, United States of America; 2 The Barshop Institute for Longevity and Aging Studies, University of Texas Health Science Center at San Antonio, San Antonio, Texas, United States of America; 3 Department of Pharmacology, University of Texas Health Science Center at San Antonio, San Antonio, Texas, United States of America; 4 Department of Cellular and Structural Biology, University of Texas Health Science Center at San Antonio, San Antonio, Texas, United States of America; 5 Department of Molecular Medicine, University of Texas Health Science Center at San Antonio, San Antonio, Texas, United States of America; 6 Geriatric Research, Education and Clinical Center and Research Service, South Texas Veterans Health Care System, San Antonio, Texas, United States of America; 7 Department of Pathology, University of California San Francisco, San Francisco, California, United States of America; 8 The Buck Institute for Age Research, Novato, California, United States of America; Università di Parma, Italy

## Abstract

**Background:**

Reduced TOR signaling has been shown to significantly increase lifespan in a variety of organisms [1], [2], [3], [4]. It was recently demonstrated that long-term treatment with rapamycin, an inhibitor of the mTOR pathway[5], or ablation of the mTOR target p70S6K[6] extends lifespan in mice, possibly by delaying aging. Whether inhibition of the mTOR pathway would delay or prevent age-associated disease such as AD remained to be determined.

**Methodology/Principal Findings:**

We used rapamycin administration and behavioral tools in a mouse model of AD as well as standard biochemical and immunohistochemical measures in brain tissue to provide answers for this question. Here we show that long-term inhibition of mTOR by rapamycin prevented AD-like cognitive deficits and lowered levels of Aβ_42_, a major toxic species in AD[7], in the PDAPP transgenic mouse model. These data indicate that inhibition of the mTOR pathway can reduce Aβ_42_ levels *in vivo* and block or delay AD in mice. As expected from the inhibition of mTOR, autophagy was increased in neurons of rapamycin-treated transgenic, but not in non-transgenic, PDAPP mice, suggesting that the reduction in Aβ and the improvement in cognitive function are due in part to increased autophagy, possibly as a response to high levels of Aβ.

**Conclusions/Significance:**

Our data suggest that inhibition of mTOR by rapamycin, an intervention that extends lifespan in mice, can slow or block AD progression in a transgenic mouse model of the disease. Rapamycin, already used in clinical settings, may be a potentially effective therapeutic agent for the treatment of AD.

## Introduction

Alzheimer's disease (AD), the most common neurodegenerative disorder in the elderly[8], is currently without effective treatment. The accumulation of soluble oligomeric forms of the amyloid-β peptide (Aβ), derived from proteolytic processing of the amyloid precursor protein (APP), is a major cause of neurotoxicity in AD[8]. The greatest known risk factor for AD is increasing age. PDAPP [also known as hAPP(J20)] mice are a well-defined mouse model of AD[9], [10]. PDAPP mice accumulate soluble and deposited Aβ and develop AD-like synaptic deficits as well as cognitive impairment and hippocampal atrophy[9], [10], [11].

The target of rapamycin (TOR) pathway is a major signaling hub that integrates nutrient/growth factor availability with cell metabolism[12] through two distinct complexes, mTORC1 and mTORC2[13]. mTORC1 functions as a nutrient/energy/redox sensor and controls protein synthesis. In addition, mTORC1 inhibits autophagy when nutrients and energy are plentiful through the phosphorylation of Unc51-like kinase 1 (ULK1) and mAtg13, the mammalian homologs of the yeast kinase Atg1 and Atg13 respectively, which are essential for the formation of pre-autophagosomal structures [14]. Phosphorylation of ULK1 and mAtg13 inhibits ULK1 activity.

mTOR also regulates autophagy through mTORC2. Active mTORC2 phosphorylates and activates Akt and PKC[15], [16]. Since Akt positively regulates mTORC1, phosphorylation of Akt by mTORC2 stimulates mTORC1 function, inhibiting autophagy. In addition, phosphorylated Akt blocks the activation of FOXO3, a member of the FOXO family of transcription factors that have a key role in lifespan extension in invertebrates. Among many targets, FOXO factors regulate expression of autophagy-related genes[17]. Thus mTORC2 activity indirectly inhibits autophagy through inactivation of FOXO3.

Autophagy is a major degradation pathway for organelles and aggregated proteins[18] such as those that cause multiple neurodegenerative diseases including AD. It has been reported that autophagy is activated in AD brains[19]. While excessive autophagic activity can lead to cell death, increased autophagy has been shown to facilitate the clearance of aggregation-prone proteins such as Aβ[20], [21], [22], pathological prion protein[23], [24], and α-synuclein[25], and to promote neuronal survival in a variety of neurodegenerative disease models. Supporting the notion that autophagy may have a protective role in AD, deletion of the beclin 1 gene in PDAPP mice impaired autophagy and resulted in large increases in Aβ levels and accelerated Aβ deposition[26]. On the other hand, the endosomal-lysosomal system is a major site of Aβ production[27], [28] and it was recently demonstated that Aβ is generated during macroautophagy both in vitro and in vivo[19]. The role of the mTOR pathway and of autophagy in AD is thus still unclear.

**Figure 1:**
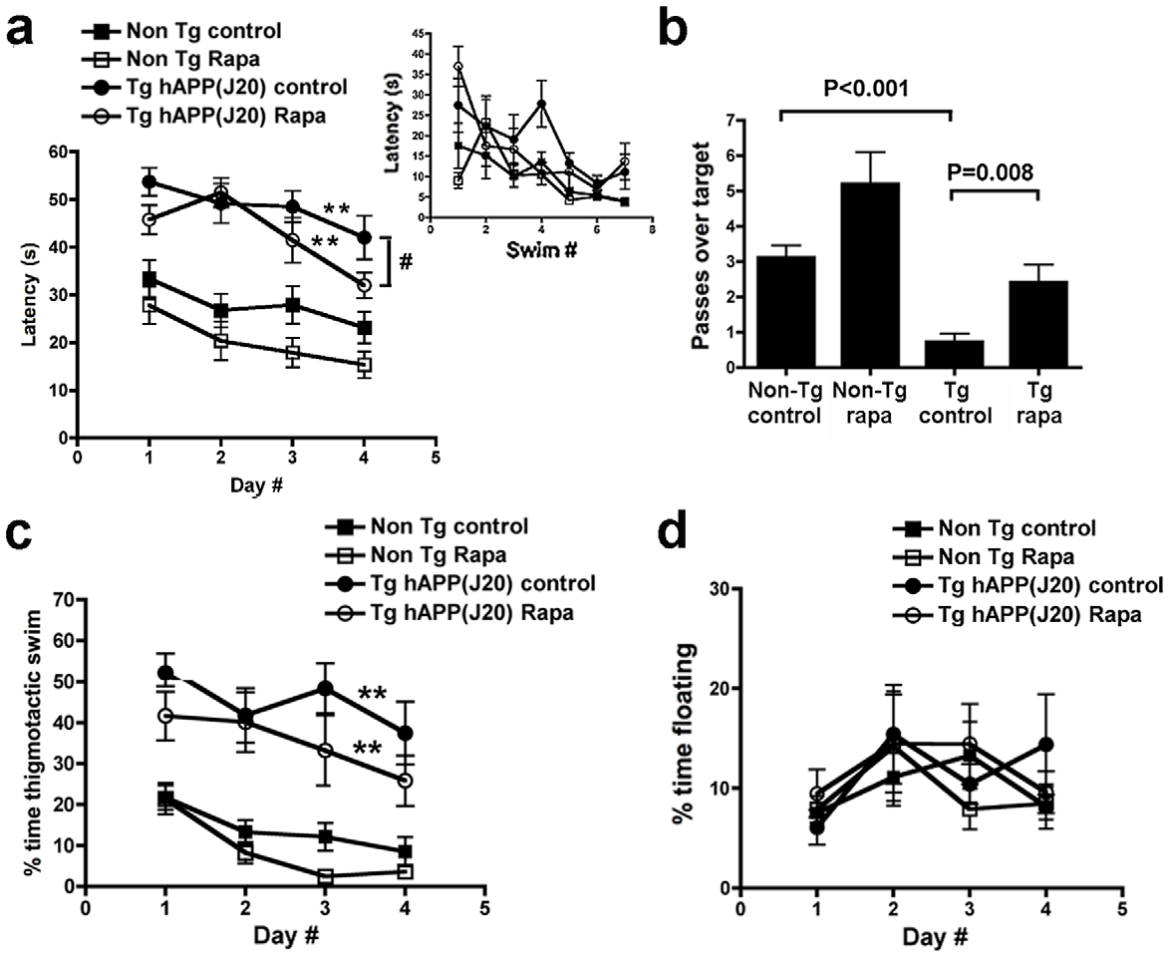
Rapamycin abrogates memory deficits in PDAPP hAPP(J20) mice. **a**, **Rapamycin improves learning in PDAPP mice**. While learning in both transgenic groups was impaired with respect to wild-type littermates' [**, *P*<0.001 for both comparisons, Bonferroni's post hoc test applied to a significant effect of genotype and treatment, *F*(3,120) = 29.46, *P*<0.0001, repeated measures two-way ANOVA], performance of rapamycin-fed PDAPP mice was improved with respect to the control-fed transgenic group only in the last day of training (^#^ *P* = 0.036 for the comparison of performance between transgenic groups, Student's *t* test), indicating improved learning of rapamycin-fed PDAPP mice at day 4. No significant interaction was observed between day number and genotype (*P* = 0.96), indicating that genotype had roughly the same effect at all times during training. Although no significant interaction was observed between day number and treatment for control-treated animals (*P* = 0.91), a significant interaction was observed between day number and treatment for rapamycin-treated groups. The effect of rapamycin treatment became more pronounced as training progressed, as indicated by the slopes for the learning curves (*m* = −5.14 for rapamycin-treated as compared to *m* = −3.58 for control-treated PDAPP transgenic mice; *m* = −4 for rapamycin-treated as compared to *m* = −2.95 for control-treated non-transgenic mice). A trend to improved learning was observed in rapamycin-treated non-Tg mice, but this difference was not significant. Overall learning was effective in all groups [*F*(3,120) = 10.29, *P*<0.0001, repeated measures two-way ANOVA]. Inset, learning was effective in all experimental groups during cued training. **b**, **Rapamycin restores spatial memory in PDAPP mice.** While retention in control-fed PDAPP mice was impaired with respect to all other groups, as previously described[11], [31], [35], [50] [*P* values are indicated, Tukey's multiple comparisons test applied to a significant effect of genotype (*P*<0.0001) in one-way ANOVA], memory in rapamycin-fed PDAPP mice was indistinguishable from that of control- or rapamycin-fed non-Tg groups. A trend to improved retention was observed in rapamycin-treated non-Tg mice, but this difference did not reach statistical significance. **c and d, Rapamycin treatment does not affect non-cognitive components of behavior.** **c**, Although transgenic groups spent more time engaged in thigmotactic swim, as described[31] (** *P*<0.001, Bonferroni's post hoc test applied to a significant effect of genotype [*F*(3,440) = 15.04, *P*<0.0001, two-way ANOVA], no significant difference in percent time spent in thigmotactic swim was observed between transgenic groups. **d**, No significant difference in floating was observed between groups. Data are mean ± SEM.

**Figure 2:**
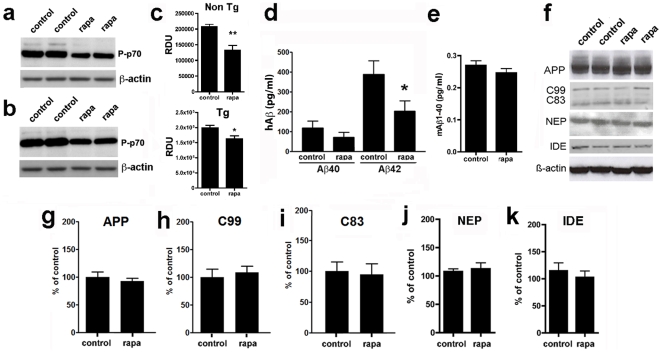
Rapamycin inhibits mTOR and decreases Aβ_42_ levels in brains of PDAPP mice. **a, b and f**, representative immunoblots of whole brain lysates from control- and rapamycin-treated PDAPP transgenic and non-transgenic littermate mice; **c, g–k**, quantitative analyses of protein or phosphoprotein levels. **a–c**, Levels of phosphorylated (activated) p70 were decreased in brains of rapamycin-treated non-transgenic (**a**) and transgenic PDAPP (**b**) mice (**c**, **, *P* = 0.006 and *, *P* = 0.01 respectively). **d**, rapamycin did not alter Aβ_40_ levels but significantly decreased soluble Aβ_42_ levels in the brains of transgenic PDAPP mice *, *P* = 0.02. Homogenates were measured at 100 mg brain tissue/ml. **e**, rapamycin did not alter levels of endogenous mouse Aβ_40_ levels in brains of non-transgenic mice. Aβ_42_ levels were below the detection limit of the ELISA (not shown). **f**, representative immunoblots of PDAPP mouse brain extracts. **g–k**, Quantitative analyses of APP, C99 and C83, NEP and IDE immunoreactivity in lysates of brains from control- and rapamycin-treated PDAPP mice. Data were normalized to β-actin levels. Student's *t* test was used to determine significance of differences between means. Data are means ± SEM.

**Figure 3:**
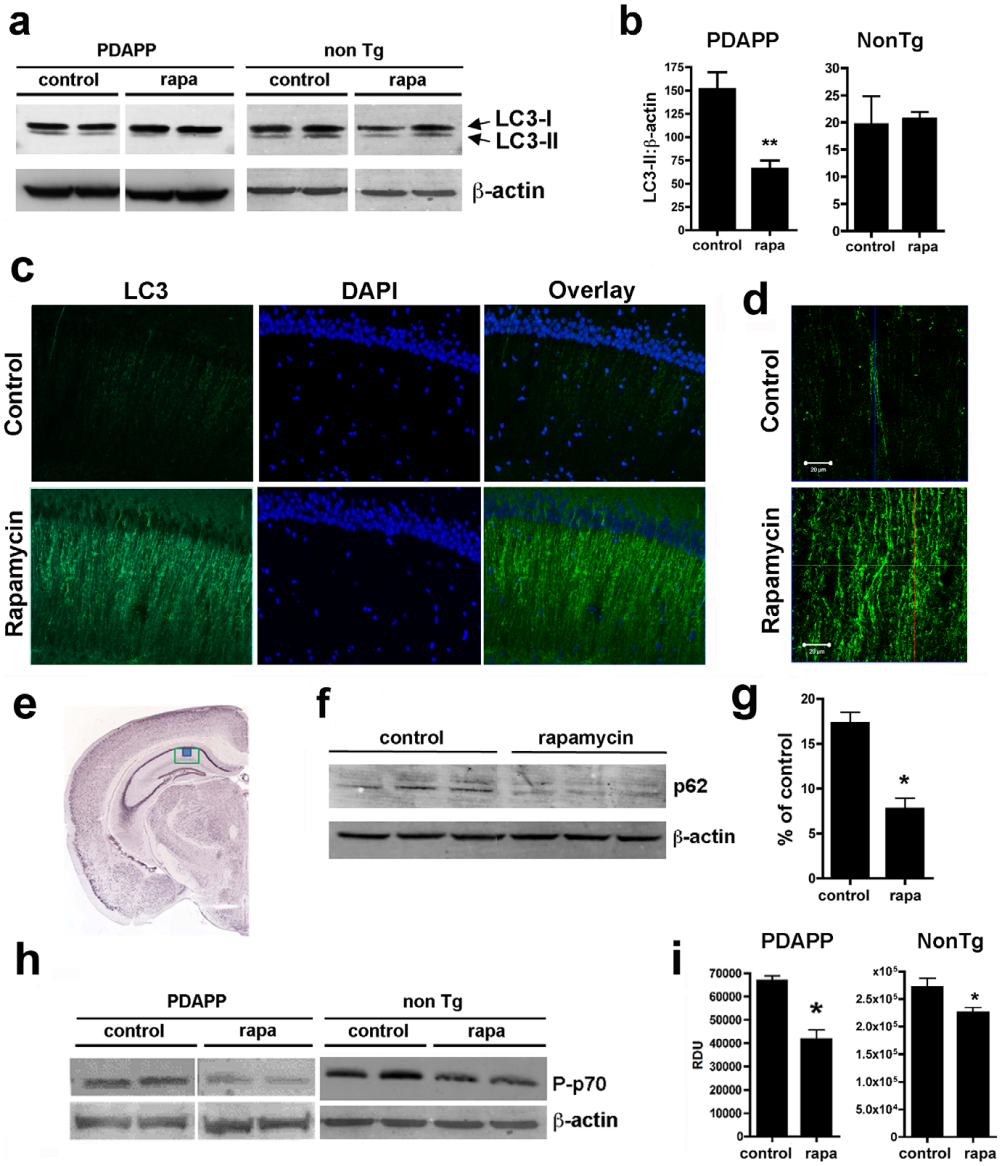
Rapamycin increases autophagy in brains of PDAPP mice. **a, f and h**, representative immunoblots of hippocampal lysates from control- and rapamycin-treated transgenic PDAPP mice and non-transgenic littermate controls. **b, g and i**, quantitative analyses. **a and b**, LC3-II levels are decreased in hippocampi of rapamycin-treated transgenic PDAPP mice (*, *P* = 0.0009), but not in hippocampi of rapamycin-treated non-transgenic littermates. **c and d**, representative epifluorescent (**c**, 200×) and higher-magnification confocal (**d**, 600×) images of hippocampal CA1 (**e**, green box, region of epifluorescent images; blue box, region of confocal images) in control- and rapamycin-fed transgenic PDAPP mice stained with an anti-LC3 antibody. An increase in LC3-immunoreactive puncta was observed in CA1 projections of transgenic PDAPP mice following rapamycin administration. **f and g**, levels of the autophagic substrate p62SQSTM are decreased (*, *P* = 0.0015) in hippocampi of rapamycin-treated PDAPP transgenic mice. **f**, representative Western blots; **g**, quantitative analyses of p62SQSTM levels. **h and i**, Levels of phosphorylated (activated) p70 were decreased in brains of rapamycin-treated PDAPP and non-transgenic mice (*, *P* = 0.001 and *P* = 0.04 respectively). Significance of differences between group means were determined using two-tailed unpaired Student's *t* test. Data are means ± SEM.

A recent report showed that long-term treatment with rapamycin, an inhibitor of the mTOR pathway[5], or ablation of the mTOR target S6K1[6] extends lifespan in mice, possibly by delaying aging. Whether inhibition of the mTOR pathway would delay or prevent age-associated disease such as AD remained to be determined. Here we show that long-term mTOR inhibition by rapamycin inhibited mTOR in brain, prevented AD-like cognitive deficits (**Fig. 1**) and lowered levels of Aβ_42_ (**Fig. 2**) in the PDAPP transgenic mouse model. These data indicate that inhibition of the mTOR pathway by long-term rapamycin treatment can reduce Aβ_42_ levels *in vivo* and block or delay AD in mice. As expected from the inhibition of mTOR, autophagy was strongly activated in hippocampus of rapamycin-treated mice. Activation of autophagy was prominent in transgenic, but not in non-transgenic, PDAPP mice (**Fig. 3**), suggesting that the reduction in Aβ and the improvement in cognitive function may be due in part to increased autophagy in neurons, possibly as a response to high levels of Aβ in transgenic mice. Consistent with this hypothesis, inhibition of mTOR by rapamycin had no effect on endogenous mouse Aβ levels in non-transgenic brains, in which the autophagic response was not activated. Thus, our data suggest that inhibition of mTOR by rapamycin, an intervention that extends lifespan in mice, can lower Aβ levels and slow or block AD progression in a transgenic mouse model of the disease, possibly by stimulating autophagy. Rapamycin, already used in clinical settings, may be a potentially effective therapeutic agent for the treatment of AD.

## Results and Discussion

A recent report showed that microencapsulated rapamycin, an inhibitor of the mTOR pathway[5], or genetic ablation of the mTOR target S6K1[6] extends lifespan in mice, possibly by retarding aging. Whether rapamycin would prevent or delay age-associated disease such as AD was unknown. To answer this question, we fed a rapamycin-supplemented diet identical to the diet that extended lifespan in mice[5] or control chow to groups of PDAPP mice and littermate non-transgenic controls for 13 weeks starting at 4 months of age. At 4 mo, hAPP(J20) PDAPP mice [9], [10], [11], [29], [30], [31], [32] show high Aβ levels and synaptic dysfunction[11], [32], but no Aβ plaques or spatial memory impairments. At the end of treatment (7 mo), learning and memory were tested using the Morris water maze [11], [31], [33], [34]. Consistent with our and others' previous observations [11], [29], [31], [32], [35], we observed significant deficits in learning and memory in control-fed transgenic PDAPP animals (**Figure 1a** and **b**). Rapamycin-fed transgenic PDAPP mice, however, showed improved learning (**Figure 1a**) and memory (**Figure 1b**), with improved performances on the last day of training and retention of the former location of the escape platform restored to levels indistinguishable from those of non-transgenic littermates (**Figure 1b**). Although no significant interactions were observed during training between day number and genotype, nor between day number and treatment for control-fed animals, a significant interaction between treatment and training day was observed, with increasingly lower latencies (thus increasingly improved performance) for rapamycin-fed PDAPP transgenic animals as training progressed (**Figure 1a**). This observation suggests that incremental learning during the acquisition phase may be accelerated or improved in rapamycin-treated PDAPP transgenic mice. A trend to improved performance during both the acquisition and testing phases was observed in rapamycin-treated non-transgenic animals, but these differences did not reach statistical significance. Thus, our data indicate that rapamycin treatment can ameliorate learning deficits and abolish memory impairments in PDAPP mice. No significant differences in thigmotaxis and floating, measures of anxiety and helplessness respectively (**Figure 1c** and **d**) nor in visual acuity (**Figure 1a**, inset) were found among groups, suggesting that improved performance in rapamycin-treated PDAPP mice is a result of effects on cognitive processes but not to effects related to non-cognitive components of behaviour, nor to differences in visual ability.

To determine the mechanism by which rapamycin treatment abolishes AD-like cognitive deficits in transgenic PDAPP mice, we examined (a) mTOR activity and (b) the proteolytic processes that generate of Aβ in PDAPP mouse brains. Phosphorylation of the mTOR target p70S6K[6] was reduced in brains of both rapamycin-treated PDAPP mice and non-transgenic littermates, indicating that mTOR activity was inhibited (**Fig. 2a–c**)[36], [37] in brains of both genotypes. Levels of Aβ_42_, but not of Aβ_40_, were reduced in rapamycin-treated transgenic PDAPP mice (**Figure 2d**). The reduction in Aβ_42_ in PDAPP brains did not likely arise from changes in production, since the abundance of C99, the C-terminal product of β-secretase cleavage of APP, as well the levels of expression of hAPP were unchanged by rapamycin (**Fig. 2f–h**). An increase in α-secretase cleavage could not explain the reduction in Aβ_42_ either, since increased α-cleavage would result in increased C83 (**Fig. 2f and i**). In addition, the reduction in Aβ_42_ did not likely arise from increased degradation, since levels of the two major Aβ-degrading activities, neprilysin (NEP)[35], and insulin-degrading enzyme (IDE)[38] were unchanged in brains of control- and rapamycin-fed PDAPP mice as well (**Fig. 2f, j–k**). Aβ deposition was not determined since no Aβ plaques are detectable in PDAPP mice at 7 mo. To determine whether levels of endogenous mouse Aβ (mAβ) would be affected by rapamycin treatment we measured mAβ_42_ and mAβ_40_ in brains of control- and rapamycin-treated non-transgenic littermates using specific ELISAs. Levels of mouse Aβ_40_ were unchanged by rapamycin treatment (**Fig. 2e**). Levels of mouse Aβ_42_ were below the limits of detection for the ELISA (please see Methods).

Autophagy is a key pathway for the clearance of aggregation-prone proteins and may have a protective role in proteinopathies[20], [39]. Inhibition of the mTOR pathway by rapamycin activates autophagy[40]. Moreover, rapamycin-induced autophagy has been implicated in the regulation of amyloid accumulation *in vivo* [26] and in the clearance of huntingtin[21], [40] and α-synuclein[41]. The role of autophagy in AD, however, is not clear[19], [27]. The induction of autophagy is associated with increased levels of microtubule-associated-protein-light-chain-3 (LC3)-II, the lipidated form of LC3[42], with respect to levels of a control protein such as β-actin or β-tubulin[43]. To determine whether rapamycin treatment affected autophagy in PDAPP brains, we examined LC3-II and β-actin in hippocampus of control- and rapamycin-treated PDAPP mice. LC3-II is created during autophagosome formation and is subsequently degraded as autophagosomes mature into autolysosomes. Lysosomal turnover of LC3-II, commonly termed autophagic flux, is the standard biochemical measurement for autophagy[43]. During autophagy, LC3-II on the cytosolic side of autophagosomal membranes is delipidated to LC3-I and is also degraded intraluminally by lysosomal hydrolases[43], [44]. Thus, decreased LC3-II levels may be observed as a consequence of robust induction of autophagic flux[43], [44]. In agreement with the expected induction of autophagy by rapamycin-mediated inhibition of mTOR, LC3-II/β-actin ratios in hippocampi of rapamycin-treated PDAPP mice were significantly decreased (**Fig. 3a–b**). In contrast, no differences in LC3-II/β-actin ratios were observed between control- and rapamycin-treated non-transgenic littermates (**Fig. 3a–b**), suggesting that rapamycin may induce autophagy as a response to high Aβ levels in hippocampi of transgenic PDAPP mice. During autophagy, LC3 redistributes to autophagosomes, which can be visualized as puncta in individual cells[19], [43], [44]. To determine whether the decreased LC3-II/β-actin ratios in hippocampi of rapamycin-treated PDAPP mice resulted from the induction of autophagic flux, we examined LC3 distribution, as well as levels of p62SQSTM, an ubiquitin-binding scaffold protein that is specifically degraded by autophagy[22], [45], in hippocampi of control- and rapamycin-treated PDAPP mice. LC3-immunoreactive puncta were increased in the projections of hippocampal neurons of rapamycin-treated PDAPP mice (**Fig. 3c–d**), suggesting that LC3 was redistributed to a vesicle-like compartment. Consistent with this observation, levels of the autophagosomal substrate p62SQSTM were significantly decreased in hippocampi of rapamycin-treated PDAPP mice (**Figure 3f–g**). Phosphorylation of p70 was significantly reduced in hippocampi of both PDAPP transgenic and non-transgenic littermate controls, indicating that mTOR activity was inhibited (**Fig. 3h–i**). Taken together, our results suggest that autophagy is induced by rapamycin-mediated mTOR inhibition specifically as a response to high Aβ levels in hippocampi of rapamycin-treated PDAPP mice.

The data presented here are, to our knowledge, the first to show that inhibition of mTOR by rapamycin decreased Aβ_42_ levels (**Fig. 2**) and rescued cognitive function (**Fig. 1**) in a mouse model of AD. Our data suggest that the reduction in Aβ_42_ levels and the improvement in cognitive function in rapamycin-treated PDAPP mice may be a consequence of the induction of autophagy in hippocampus (**Fig. 3**) by high levels of Aβ in PDAPP transgenic brains. Consistent with a key role for high levels of Aβ in the activation of autophagy when mTOR activity is reduced, rapamycin did not induce autophagy in brains of rapamycin-treated non-transgenic mice, in which levels of endogenous Aβ are much lower than those in PDAPP transgenic brains. In addition, rapamycin treatment did not induce autophagy and did not affect levels of endogenous Aβ in non-transgenic mice, suggesting that autophagy may have a key role in reducing Aβ_42_ in transgenic PDAPP brains. Rapamycin was administered to PDAPP mice at a dose that was previously shown to extend lifespan in mice[5]. Our observations are thus consistent with a recent report that showed that the life-extending effect of TOR inhibition in *C. elegans* requires autophagy[46]. It is possible that the activation of autophagy as a response to Aβ accumulation is reduced with increasing age[47], [48]. This may be a consequence of inactivation of DAF family member FOXO factors by mTOR signaling during aging[13]. Prolonged rapamycin treatment may thus release mTOR-mediated inhibition of autophagy and allow for the reduction of Aβ levels through this clearance mechanism in transgenic PDAPP brains. Although rapamycin treatment did not activate autophagy nor reduce endogenous mouse Aβ levels, it inhibited mTOR function in non-transgenic littermate brains, and this group showed trends to improved learning and retention. Although the differences in performance between control-fed and rapamycin-fed non-transgenic groups were not significant, they may suggest that changes in pathways different from autophagy (such as effects on the regulation of protein synthesis) as a result of long-term mTOR inhibition may have a positive effect on learning and memory. Reducing Aβ levels abolishes cognitive impairments in a variety of models[49]. We cannot rule out, however, mTOR-dependent effects on cognition that may be additive to the benefit of reduced Aβ in our model system.

In summary, our data suggest that inhibition of mTOR by rapamycin[5], an intervention that extends lifespan in mice[5], [6], can slow or block AD progression in a transgenic mouse model of the disease. Rapamycin, already used in clinical settings, may thus be a potentially effective therapy for the prevention or treatment of AD.

## Methods

### Mice

The derivation and characterization of PDAPP [hAPP(J20)] mice has been described elsewhere[9], [10], [29]. PDAPP mice were maintained by heterozygous crosses with C57BL/6J mice (Jackson Laboratories, Bar Harbor, ME). PDAPP mice were heterozygous with respect to the transgene. Non-transgenic littermates were used as controls. Rapamycin administration and behavioral experiments involving PDAPP mice were conducted at the Buck Institute for Age Research, Novato, CA. Experimental groups were: control-fed non-Tg, n = 10; rapamycin-fed non-Tg, n = 10; control-fed Tg, n = 12; rapamycin-fed Tg, n = 12, all animals were males and 7 month-old at the time of testing. Rapamycin was administered for 13 weeks starting at 4 months of age.

### Rapamycin treatment

Mice were fed chow containing either microencapsulated rapamycin at 2.24 mg/kg or a control diet as described by Harrison et al.[5]. Rapamycin was used at 14 mg per kg food (verified by HPLC). On the assumption that the average mouse weighs 30 gm and consumes 5 gm of food/day, this dose supplied 2.24 mg rapamycin per kg body weight/day[5]. All mice were given *ad libitum* access to rapamycin or control food and water for the duration of the experiment. Body weights and food intake were measured weekly. Food consumption remained constant for both control- and rapamycin-fed groups during treatment (no significant effect of week # on food consumption by two-way ANOVA, *P* = 0.108). Food consumption was higher for rapamycin-fed animals (by an average of 2.12±0.22 g/mouse/week at all times during the experiment (*P*<0.001, two-way ANOVA). This may be a result of the inhibition of the mTOR pathway, which is expected to mimic the unfed state by decreasing mTOR activity. Littermates (transgenic and non-transgenic mice) were housed together, thus we could not distinguish effects of genotype on food consumption. In spite of the differences in food consumption, overall body weight of control- and rapamycin-fed groups was not significantly different (25.59±0.43 to 26.89±0.44 for control-fed and 26.77±0.48 to 28.11±0.53 for rapamycin-fed animals) although body weight increased moderately for both groups during the 13 week treatment, possibly as a result of the change in base chow composition (increases were 5% and 11% for rapamycin-fed transgenic and non-transgenic groups respectively; 10 and 15% for control-fed transgenic and non-transgenic groups respectively). The relatively higher increase in body weight for non-transgenic animals is not unexpected, since non-transgenic animals tend to be slightly (1**–**3 g) heavier than PDAPP transgenic mice.

### Behavioral testing

The Morris water maze (MWM)[11], [31], [33] was used to test spatial memory. All animals showed no deficiencies in swimming abilities, directional swimming or climbing onto a cued platform during pre-training and had no sensorimotor deficits as determined with a battery of neurobehavioral tasks performed prior to testing. All groups were assessed for swimming ability 2 days before testing. The procedure described by Morris et al.[33] was followed as described[11], [31]. Briefly, transgenic and non-transgenic PDAPP mice were given a series of 6 trials, 1 hour apart in a light-colored tank filled with opaque water whitened by the addition of non-toxic paint at a temperature of 24.0±1.0°C. In the visible portion of the protocol, animals were trained to find a 12×12-cm submerged platform (1 cm below water surface) marked with a colored pole that served as a landmark placed in different quadrants of the pool. The animals were released at different locations in each 60-second trial. If mice did not find the platform in 60 seconds, they were gently guided to it. After remaining on the platform for 20 seconds, the animals were removed and placed in a dry cage under a warm heating lamp. Twenty minutes later, each animal was given a second trial using a different release position. This process was repeated a total of 6 times for each mouse, with each trial ∼20 minutes apart. In the non-cued part of the protocol, the water tank was surrounded by opaque dark panels with geometric designs at approximately 30 cm from the edge of the pool, to serve as distal cues. The animals were trained to find the platform with 6 swims/day for 4 days following the same procedure described above. At the end of training, a 30-second probe trial was administered in which the platform was removed from the pool. The number of times that each animal crossed the previous platform location was determined as a measure of platform location retention. During the course of testing, animals were monitored daily, and their weights were recorded weekly. Performance in all tasks was recorded by a computer-based video tracking system (Water2020, HVS Image, U.K). Data were analyzed offline by using HVS Image and processed with Microsoft Excel.

### Western blotting and Aβ determinations

Mice were euthanized by isoflurane overdose followed by cervical dislocation. Hemibrains were flash frozen. One hemibrain was homogenized in liquid N_2_ while the other was used in immunohistochemical determinations (5–6 per group) and for hippocampal dissections (5–6 per group). Half brains were microdissected to isolate the hippocampus by peeling away the cortex from the underlying hippocampus and releasing the hippocampus from the surrounding tissue, in particular the fimbria, by using fine surgical tweezers (Fine Science Tools) and then lifting toward the midline. The hippocampus separates easily but does include some adjacent white matter, which is then carefully tweezed off. We also obtained hippocampal tissue from at least 12×10 µm unfixed frozen sections mounted on glass slides. All but the hippocampal area was removed using a scalpel under a SZ60 Olympus dissecting microscope. The hippocampal tissue itself was removed by beading 10 µl of RIPA lysis buffer (25 mM Tris-HCl, pH 7.6; 150 mM NaCl; 1% NP40; 1% sodium deoxycholate; 0.1% sodium dodecyl sulfate) on it and then pipetting it up. For Western blot analyses, proteins from soluble fractions of brain LN_2_ homogenates and from hippocampal dissections were resolved by SDS/PAGE (Invitrogen, Temecula, CA) under reducing conditions and transferred to a PVDF membrane, which was incubated in a 5% solution of non-fat milk or in 5% BSA for 1 hour at 20°C. After overnight incubation at 4°C with primary antibodies, the blots were washed in TBS-Tween 20 (TBS-T) (0.02% Tween 20, 100 mM Tris pH 7.5; 150 nM NaCl) for 20 minutes and incubated at room temperature with appropiate secondary antibodies. The blots were then washed 3 times for 20 minutes each in TBS-T and then incubated for 5 min with Super Signal (Pierce, Rockford, IL), washed again and exposed to film or imaged with a Typhoon 9200 variable mode imager (GE Healthcare, NJ). Human Aβ_40_ and Aβ_42_ levels, as well as endogenous mouse Aβ_40_ levels were measured in guanidine brain homogenates using specific sandwich ELISA assays (Invitrogen, Carlsbad, CA) as described[11].

### Antibodies

Antibodies used were: anti-IDE (Abcam, ab32216); anti-NEP (R&D AF1126); anti-APP (CT15 (REF); anti-phospho-p70 (Cell Signaling, #9206); anti-β-actin (Sigma, A3853); anti-LC3 (Novus Biologicals, NB100-2331); anti-p62 (Progen, GP62-C).

### Immunohistochemistry

Ten-micrometer coronal cryosections from snap-frozen brains were post-fixed in 4% paraformaldehyde and stained with LC3-specific antibodies (10 µg/ml, Nous, Littleton, CO) followed by AlexaFluor488-conjugated donkey anti-rabbit IgG (1∶500, Molecular Probes, Invitrogen, CA), and imaged with a epifluorescence microscope (Nikon Eclipse E800 with a FITC cube) or with a laser scanning confocal microscope (Zeiss LSM 510) using a 488 Argon laser and a 505 long-pass filter. Images were obtained using 20× and 60× objectives. Z-stacks of confocal images were processed using LSM Viewer software (Zeiss). All images were collected in the stratum radiatum of the hippocampus immediately beneath the CA1 layer at Bregma ∼−2.18. The MBL Mouse Brain Atlas was used for reference.

### Statistical analyses

Statistical analyses were performed using GraphPad Prism (GraphPad, San Diego, CA) and StatView. In two-variable experiments, two-way ANOVA followed by Bonferroni's *post-hoc* tests were used to evaluate the significance of differences between group means. When analyzing one-variable experiments with more than 2 groups, significance of differences among means was evaluated using one-way ANOVA followed by Tukey's *post-hoc* test. Evaluation of differences between two groups was evaluated using Student's *t* test. Values of *P*<0.05 were considered significant.
