# Correction: Inhibition of mTOR by Rapamycin Abolishes Cognitive Deficits and Reduces Amyloid-β Levels in a Mouse Model of Alzheimer's Disease

**DOI:** 10.1371/annotation/05c1b976-7eab-4154-808d-0526e604b8eb

**Published:** 2011-11-18

**Authors:** Patricia Spilman, Natalia Podlutskaya, Matthew J. Hart, Jayanta Debnath, Olivia Gorostiza, Dale Bredesen, Arlan Richardson, Randy Strong, Veronica Galvan

The affiliation for the first author was incorrect. Patricia Spilman is not affiliated with #8 but with #1 Department of Physiology, University of Texas Health Science Center at San Antonio, San Antonio, Texas, United States of America, and #2 The Barshop Institute for Longevity and Aging Studies, University of Texas Health Science Center at San Antonio, San Antonio, Texas, United States of America. 

