# Correction: Inhibition of mTOR by Rapamycin Abolishes Cognitive Deficits and Reduces Amyloid-β Levels in a Mouse Model of Alzheimer's Disease

**DOI:** 10.1371/annotation/85294f34-4d64-4189-b4c8-b1d77c0b3908

**Published:** 2011-12-27

**Authors:** Patricia Spilman, Natalia Podlutskaya, Matthew J. Hart, Jayanta Debnath, Olivia Gorostiza, Dale Bredesen, Arlan Richardson, Randy Strong, Veronica Galvan

The correction to Patricia Spilman's affiliation was in error: no correction was needed. Patricia Spilman is affiliated with #8, The Buck Institute for Age Research, Novato, California, United States of America, not with #1 or #2. 

